# 1414. Residual Surface Disinfectant Reduces Finger Transfer of Antibiotic Resistant Bacteria and Viruses from Surfaces for 24 hours after Treatment

**DOI:** 10.1093/ofid/ofad500.1251

**Published:** 2023-11-27

**Authors:** Charles P Gerba

**Affiliations:** University of Arizona, Tucson, Arizona

## Abstract

**Background:**

It is believed that as much as 80% of common infections are spread by the hands. Touching surfaces (fomites) contaminated with infectious microorganisms results in their transfer to the hands (fingers). Subsequent touching of the mouth, nose, or skin can result in their transmission. Disinfection of surfaces has been found to reduce such transmission. Unfortunately, highly touched surfaces (door knobs, escalator hand rails, buttons, etc.) can quickly become re-contaminated. Disinfectants which leave a residual can act as an additional barrier in between regular disinfection of surfaces, reducing the risk of transmission.

The goal of this study was to assess the reduction in the transfer of bacteria and viruses from surfaces treated with a quaternary ammonium based disinfectant (Actizone F5® spray) that leaves a residual disinfectant capability for more than 24 hours.

**Methods:**

Stainless steel and plastic coupons were treated with Actizone F5® spray and allowed to dry for 24 hours. The test microorganisms were then added to the surfaces and after 2, 5 or 10 minutes, the surface was touched with a finger. The number of microorganisms transferred to the finger was then quantified. The experiment was repeated with untreated coupons as a control.

**Results:**

The degree of finger transfer was reduced by more than 99.99% by the treated coupons for *Salmonella* Typhimurium, MRSA, *Acinetobacter baumannii*, and *Escherichia coli*. *Candida auris* was reduced by > 99.99% on the stainless steel and ∼99.6% for the plastic coupons. *Pseudomonas aeruginosa* and antibiotic-resistant *Klebsiella pneumoniae* (CRE) were reduced by ∼98%. The transfer of human coronavirus 229E was reduced by 98.7% from the stainless steel surfaces.

**Conclusion:**

Transfer of all the test microorganisms was reduced by the treated surfaces even after 24 hours, demonstrating residual activity. The transfer for some of the microorganisms was less from the ABS plastic than from the stainless steel surfaces. This may have been due to the attachment or spreading of the product on this type of surface, resulting in a less even coating. A longer contact time may result in significant reduction of these microorganisms. The relative humidity and short exposure time to the treated surfaces used in this study should probably be considered a worst case scenario.

**Disclosures:**

**Charles P. Gerba, PhD**, □ Corning: Advisor/Consultant| □ Reckitt: Advisor/Consultant| □ Reckitt: Grant/Research Support| □ Savory: Grant/Research Support

